# Kobuvirus in Domestic Sheep, Hungary

**DOI:** 10.3201/eid1605.091934

**Published:** 2010-05

**Authors:** Gábor Reuter, Ákos Boros, Péter Pankovics, László Egyed

**Affiliations:** ÁNTSZ Regional Institute of State Public Health Service, Pécs, Hungary (G. Reuter, Á. Boros, P. Pankovics); Veterinary Medical Research Institute of the Hungarian Academy of Sciences, Budapest, Hungary (L. Egyed)

**Keywords:** Kobuvirus, sheep, picornavirus, viruses, Hungary, letter

**To the Editor**: Picornaviruses (family *Picornaviridae*) are small, nonenveloped viruses with single-stranded, positive-sense genomic RNA. They are divided into 12 genera: *Enterovirus, Aphthovirus, Cardiovirus, Hepatovirus, Parechovirus, Erbovirus, Teschovirus, Sapelovirus, Senecavirus, Tremovirus, Avihepatovirus*, and *Kobuvirus*. The genus *Kobuvirus* consists of 2 officially recognized species, *Aichi virus* (*1*) and *Bovine kobuvirus* (*2*), and 1 candidate species, porcine kobuvirus (*3*). The kobuvirus genome is ≈8.2–8.4 kb long and has the typical picornavirus genome organization of leader (L) protein following the structural (viral protein [VP] 0, VP3, and VP1) and nonstructural (2A–2C and 3A–3D) regions (*2*,*4*). The genetic identity on the coding region between Aichi (strain A846/88), bovine (U-1), and porcine (S-1-HUN) viruses is between 35% (L protein) and 74% (3D region) (*2*,*4*).

Aichi virus and bovine kobuvirus were first detected in fecal samples from humans and cattle in Japan, in 1991 and 2003, respectively (*1*,*2*). Porcine kobuvirus was identified from domestic pigs in Hungary in 2008 (*3*,*4*). Recent studies demonstrated that Aichi virus circulates in Asia (*5*), Europe (*6*,*7*) including Hungary (*4*), South America (*6*), and North Africa (*8*) and can cause gastroenteritis in humans. In addition, bovine and porcine kobuviruses are detected among these farm animals in Europe (*4*) and Asia (*2*,*9*). These data indicate that kobuviruses are widely distributed geographically and raise the possibility of additional animal host species. We detected kobuvirus in sheep.

On March 17, 2009, a total of 8 fecal samples were collected from young, healthy, domestic sheep (*Ovis aries*) <3 weeks of age in a herd of 400 animals in central Hungary. At this farm, merino ewes from Hungary were mated with blackhead meat rams from Germany. At the time of sampling, no clinical signs of diarrhea were reported. Reverse transcription–PCR was performed by using generic kobuvirus screening primers (UNIV-kobu-R/F) reported previously (*4*). These primers were designed for Aichi virus (GenBank accession no. AB040749), bovine (AB084788), and porcine kobuvirus (EU787450) sequences and amplify a 216-nt region of 3D (RNA-dependent RNA polymerase region). The continuous 3D and 3′ untranslated regions (UTRs) of the kobuvirus genome in sheep were determined by using the 5′/3′ RACE (rapid amplification of cDNA ends) kit, 2nd generation (Roche Diagnostics GmbH, Mannheim, Germany) and primers UNIV-kobu-F and S-1-F-7518/7540 (5′-CACTTCCATCATCAACACCATCA-3′ corresponding to nt 7518–7540 of bovine kobuvirus) (*4*). PCR products were sequenced directly in both directions by using the BigDye Reaction Kit (Applied Biosystems, Warrington, UK) with the PCR primers and sequenced by an ABI PRISM 310 Genetic Analyzer (Applied Biosystems, Stafford, TX, USA). Phylogenetic analysis was conducted by using MEGA version 4.1 (www.megasoftware.net). The sequence for kobuvirus/sheep/TB3-HUN/2009/Hungary was submitted to GenBank under accession no. GU245693.

Of the 8 sheep fecal samples, 5 (62.5%) were positive for kobuvirus. The partial 3D region (216 nt) was genetically identical for all 5 strains. The 3′ continuous nucleotide sequence of the partial 3D (688 nt) and 3′ UTR (174 nt) regions of strain kobuvirus/sheep/TB3-HUN/2009/Hungary (TB3-HUN; GU245693) was determined. TB3-HUN had 59%–66% (862) nt and 77%–84% aa identities to Aichi and porcine kobuviruses, respectively. Strain TB3-HUN had 89/97% nt/aa and 86% nt identities to bovine kobuvirus in the 3D/3′ UTR (862 nt) and 3′ UTR (174 nt) regions, respectively. Phylogenetic analysis of the overlapping partial 3D/3′ UTR nucleotide sequence of TB3-HUN from sheep and of reference bovine, porcine, and human kobuviruses confirmed that ovine kobuvirus strain TB3-HUN is related to bovine kobuviruses (Figure).

**Figure:**
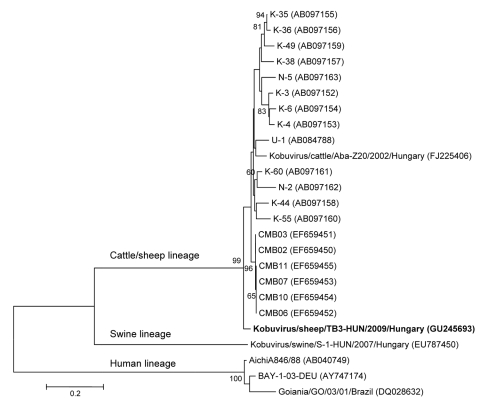
Phylogenetic analysis of kobuvirus in sheep (kobuvirus/sheep/TB3-HUN/2009/Hungary, GU245693) and kobuvirus lineages in humans, cattle, and swine, according to the 862-nt fragment of the kobuvirus 3D/3′ untranslated regions. The phylogenetic tree was constructed by using the neighbor-joining clustering method with distance calculation and the maximum-composite likelihood correction for evolutionary rate with MEGA version 4.1 software (www.megasoftware.net). Bootstrap values (based on 1,000 replicates) are given for each node if >50%. Reference strains were obtained from GenBank. **Boldface** indicates virus detected in sheep. Scale bar indicates nucleotide substitutions per site.

The nucleotide sequence of the partial 3D/3′ UTR region of kobuvirus in sheep has high nucleotide identity to bovine kobuviruses and forms the same lineage (but a different sublineage) with the kobuvirus strains in cattle. This result raised the following questions: can a highly similar kobuvirus be present in (and pathogenic for) 2 animal species (cattle and sheep), or is this result a consequence of natural contamination? The concept of sheep as host is supported by the high prevalence of kobuvirus in young healthy sheep; by the sublineage position of the sheep strain on the phylogenetic tree according to the most conserved genetic region; and by the genetic relation between the 2 potential ruminant hosts, cattle and sheep. The existence of 1 pathogen in 2 host species (cattle and sheep) is well known, e.g., for bluetongue virus, adenoviruses, ovine herpesvirus type 2, and foot-and-mouth disease picornaviruses (*10*). Alternatively, the possibility of natural contamination cannot be excluded. The possibility of passive virus shedding in sheep exists because a cattle farm was located next to the tested sheep herd and would enable fecal–oral transmission of kobuvirus between these farm animals. Both possibilities (host and passive virus reservoir) are preliminary perceptions, regardless which is true. Further molecular and epidemiologic studies are required to determine the relevance, distribution, and diversity of kobuvirus or kobuviruses in sheep.

## References

[1] Yamashita T, Kobayashi S, Sakae K, Nakata S, Chiba S, Ishihara Y, Isolation of cytopathic small round viruses with BS-C-1 cells from patients with gastroenteritis. J Infect Dis. 1991;164:954–7. 1658159 10.1093/infdis/164.5.954

[2] Yamashita T, Ito M, Kabashima Y, Tsuzuki H, Fujiura A, Sakae K. Isolation and characterization of a new species of kobuvirus associated with cattle. J Gen Virol. 2003;84:3069–77. 10.1099/vir.0.19266-0 14573811

[3] Reuter G, Boldizsár Á, Kiss I, Pankovics P. Candidate new species of *Kobuvirus* in porcine hosts. Emerg Infect Dis. 2008;14:1968–70. 10.3201/eid1412.080797 19046542 PMC2634637

[4] Reuter G, Boldizsár Á, Pankovics P. Complete nucleotide and amino acid sequences and genetic organization of porcine kobuvirus, a member of a new species in genus *Kobuvirus*, family *Picornaviridae.* Arch Virol. 2009;154:101–8. 10.1007/s00705-008-0288-2 19096904

[5] Pham NT, Khamrin P, Nguyen TA, Kanti DS, Phan TG, Okitsu S, Isolation and molecular characterization of Aichi viruses from fecal specimens collected in Japan, Bangladesh, Thailand, and Vietnam. J Clin Microbiol. 2007;45:2287–8. 10.1128/JCM.00525-07 17522267 PMC1932998

[6] Oh DY, Silva PA, Hauroeder B, Deidrich S, Cardoso DD, Schreier E. Molecular characterization of the first Aichi viruses isolated in Europe and in South America. Arch Virol. 2006;151:1199–206. 10.1007/s00705-005-0706-7 16421634

[7] Ambert-Balay K, Lorrot M, Bon F, Giraudon H, Kaplon J, Wolfer M, Prevalence and genetic diversity of Aichi virus strains in stool samples from community and hospitalized patients. J Clin Microbiol. 2008;46:1252–8. 10.1128/JCM.02140-07 18256215 PMC2292896

[8] Sdiri-Loulizi K, Gharbi-Khélifi H, de Rougemont A, Chouchane S, Sakly N, Ambert-Balay K, Acute infantile gastroenteritis associated with human enteric viruses in Tunisia. J Clin Microbiol. 2008;46:1349–55. 10.1128/JCM.02438-07 18287312 PMC2292941

[9] Khamrin P, Maneekarn N, Kongkaew A, Kongkaew S, Okitsu S, Ushijima H. Porcine kobuvirus in piglets, Thailand. Emerg Infect Dis. 2009;15:2075–6. 10.3201/eid1512.090724 19961712 PMC3044534

[10] Thiry E. Clinical virology of ruminants. Paris: Wolters-Kluwer; 2007.

